# Patients with Minimal Hepatic Encephalopathy Show Altered Thermal Sensitivity and Autonomic Function

**DOI:** 10.3390/jcm10020239

**Published:** 2021-01-11

**Authors:** Dalia Rega, Mika Aiko, Nicolás Peñaranda, Amparo Urios, Juan-José Gallego, Carla Giménez-Garzó, Franc Casanova, Alessandra Fiorillo, Andrea Cabrera-Pastor, Teresa San-Miguel, Cristina Ipiens, Desamparados Escudero-García, Joan Tosca, Cristina Montón, María-Pilar Ballester, José Ballester, Luis Aparicio, María-Pilar Ríos, Lucía Durbán, Amparo Mir, Elena Kosenko, Paula Cases, Vicente Felipo, Carmina Montoliu

**Affiliations:** 1 INCLIVA, Health Research Institute, 46010 Valencia, Spainaurios@incliva.es (A.U.); jjgallego@incliva.es (J.-J.G.); fcasanova@incliva.es (F.C.); afiorillo@incliva.es (A.F.); acabrera@incliva.es (A.C.-P.); Mariapi_28_@hotmail.com (M.-P.B.); 2 Servicio de Neurofisiología, Hospital Clínico de Valencia, 46010 Valencia, Spain; aiko_mik@gva.es (M.A.); nikopenaranda@gmail.com (N.P.); ipiens_cri@gva.es (C.I.); cases_pau@gva.es (P.C.); 3 Laboratorio de Neurobiología. Centro Investigación Príncipe Felipe, 46012 Valencia, Spain; cgimenez@cipf.es (C.G.-G.); vfelipo@cipf.es (V.F.); 4 Departamento de Patología, Facultad de Medicina, Universidad de Valencia, 46010 Valencia, Spain; Teresa.Miguel@uv.es; 5 Servicio de Medicina Digestiva, Hospital Clinico de Valencia, 46010 Valencia, Spain; m.desamparados.escudero@uv.es (D.E.-G.); jtosca469@cv.gva.es (J.T.); monton_cri@gva.es (C.M.); pballesterf@yahoo.es (J.B.); 6 Departamento de Medicina, Universidad de Valencia, 46010 Valencia, Spain; amparo.mir@uv.es; 7 Departamento de Anatomía y Embriología, Universidad Valencia, 46010 Valencia, Spain; luis.aparicio@uv.es; 8 Servicio de Digestivo, Hospital Arnau de Vilanova, 46015 Valencia, Spain; mriosp73@hotmail.com (M.-P.R.); durban_luc@gva.es (L.D.); 9 Institute of Theoretical and Experimental Biophysics of Russian Academy of Sciences, 142290 Pushchino, Russia; eakos@rambler.ru

**Keywords:** minimal hepatic encephalopathy, nerve conduction, thermal sensitivity, autonomic testing

## Abstract

Cirrhotic patients may experience alterations in the peripheral nervous system and in somatosensory perception. Impairment of the somatosensory system could contribute to cognitive and motor alterations characteristic of minimal hepatic encephalopathy (MHE), which affects up to 40% of cirrhotic patients. We assessed the relationship between MHE and alterations in thermal, vibration, and/or heat pain sensitivity in 58 cirrhotic patients (38 without and 20 with MHE according to Psychometric Hepatic Encephalopathy Score) and 39 controls. All participants underwent attention and coordination tests, a nerve conduction study, autonomic function testing, and evaluation of sensory thresholds (vibration, cooling, and heat pain detection) by electromyography and quantitative sensory testing. The detection thresholds for cold and heat pain on the foot were higher in patients with, than those without MHE. This hyposensitivity was correlated with attention deficits. Reaction times in the foot were longer in patients with, than without MHE. Patients with normal sural nerve amplitude showed altered thermal sensitivity and autonomic function, with stronger alterations in patients with, than in those without MHE. MHE patients show a general decrease in cognitive and sensory abilities. Small fibers of the autonomic nervous system and thermal sensitivity are altered early on in MHE, before large sensory fibers. Quantitative sensory testing could be used as a marker of MHE.

## 1. Introduction

Hepatic encephalopathy (HE) is defined as a ‘brain dysfunction caused by liver insufficiency and/or portosystemic shunting’ [1]. HE may be classified as covert or overt. The terms minimal or covert hepatic encephalopathy are used when cognitive and motor alterations induced by liver cirrhosis are not evident but may be unveiled using psychometric or neurophysiological tests. Overt hepatic encephalopathy is applied when neurological alterations are more evident. The scale most often used for grading the extent of HE is the West Haven criteria, which distinguishes between four grades of clinically overt HE. Currently, some experts suggest differentiating between covert HE (MHE plus grade I HE according to West Haven criteria) and overt HE (grades II–IV) [1].

Around 30–50% of cirrhotic patients present minimal hepatic encephalopathy (MHE), characterized by attention deficits, psychomotor slowing, and mild cognitive impairment, which impair quality of life and reduce life span [1,2,3,4,5,6]. MHE patients have difficulty performing everyday tasks such as driving [7,8]. They also have higher risk of falls, accidents, and hospitalization, creating a greater economic burden on the health and social care systems [9,10,11]. MHE is undetectable by traditional clinical methods and is currently diagnosed with a battery of psychometric tests called the psychometric hepatic encephalopathy score (PHES) [12,13]. Although these psychometric tests are a first-line tool for diagnosing MHE, several reports indicate a subset of cirrhotic patients who show mild cognitive and motor deficits not detected by the PHES [14,15,16,17,18].

Cirrhotic patients may show alterations in the peripheral nervous system, for example in somatosensory perception [19]. Somatosensory perception is composed of different modalities, which can be grouped in three subsystems. The first collects information from cutaneous mechanoreceptors such as fine touch, pressure, and vibration. The second subsystem receives proprioceptive information from specialized receptors associated with the muscles, tendons, and articulations, called proprioceptors. The third system derives from receptors that inform of pain, temperature changes, and gross touch. All these data are conducted through afferent fibers of pseudounipolar neurons of the spinal ganglion, reaching the posterior spinal medulla, where crossing may take place directly (protopathic sensibility) or at the brain stem (epicritic sensibility). Finally, the afferents end in a contralateral manner to the location of the received stimulus, in the corresponding somatotopic region of the cortex.

The autonomic nervous system controls the functioning of the different body systems within the organism. This system, through its three efferent components—sympathetic, parasympathetic, and enteric—innervates the heart muscle, the smooth muscle of all organs, and the exocrine and endocrine glands. The most frequent clinical manifestations of autonomic dysfunctions are cardiovascular, digestive, sudomotor, ocular, genitourinary…, and there are several functional tests to evaluate autonomic function, such as the assessment of the cardiovascular system (RR interval, valsalva maneuver,…), and the sudomotor system, among others [20].

Clinically, polyneuropathy is defined by a series of motor, sensory, and autonomic symptoms. To diagnose peripheral polyneuropathy and classify the type of fiber and modality affected (small or large fibers, sensory, motor, or both) sensory and motor nerve conduction studies are performed, which can often identify the primary pathological process.

Several sensory nerve conduction studies have shown that up to 70% of cirrhotic patients could be affected by sensorimotor peripheral neuropathy [19,21]. Polyneuropathy associated with liver cirrhosis is included within the chronic axonal polyneuropathies of toxic-metabolic origin, characterized by length-dependent or dying-back axon degeneration, initially affecting the sensory fibers and with a pattern of distal predominance [22]. Some chronic axonal polyneuropathies with predominantly sensory or autonomic alterations may affect only a small caliber axonal fibers, potentially undetectable in nerve conduction studies. These small caliber fibers include sympathetic and parasympathetic unmyelinated fibers that are involved in conducting pain and temperature sensation [20,23]. Alteration of these fibers can be evaluated by autonomic electrophysiologic studies such as electrodermal activity related to sweating (sympathetic skin response), heart rate variability (RR interval measurement) and thermotesting.

Previous studies using neurophysiological methods have found impairments in both central and peripheral parts of the somatosensory system in patients with liver cirrhosis and overt HE [24].

Analyzing somatosensory system function is of interest in MHE patients because this system is involved in awareness, attention, and motor response [25], areas which are altered in these patients. Impaired function of the somatosensory system could contribute to the development of mild cognitive and motor deficiencies in MHE. This theory is supported by sensory deficits manifested in patients with cognitive impairment-associated diseases such as Alzheimer’s and Parkinson’s [26,27].

Mild cognitive impairment was linked to early changes in the primary somatosensory cortex, which has been proposed as a sensitive marker for cognitive decline [26].

A better characterization of sensory perception, nerve conduction, and autonomic function could enable detection of MHE at earlier stages and with greater sensitivity. The aim of this study was to evaluate and characterize thermal, vibration and heat pain sensitivity in cirrhotic patients and healthy controls, and to assess any differences between patients with and without MHE. We evaluated different neurological functions such as attention, concentration, mental processing speed, working memory, and bimanual and visuomotor coordination, using specific psychometric tests. These functions are altered at early stages of MHE [14]. We assessed the correlations of performance in these functions with alterations in sensory perception and autonomic function. We also performed sensory and motor nerve conduction studies and autonomic function testing to classify the type of fiber affected (small or large fibers, sensory, motor, or both), and pinpoint the primary pathological process.

## 2. Experimental Section

### 2.1. Patients and Controls

Fifty-eight patients with liver cirrhosis were consecutively recruited from outpatient clinics of Clinico and Arnau de Vilanova hospitals in Valencia, Spain. Cirrhosis diagnosis was based on clinical, biochemical, and ultrasonographic data. The exclusion criteria were overt hepatic encephalopathy, recent alcohol intake (<6 months), infection, recent antibiotic use or gastrointestinal bleeding (<6 weeks), recent use of drugs affecting cognitive function (<6 weeks), presence of hepatocellular carcinoma, and neurological or psychiatric disorders. Patients with insulin-dependent diabetes were also excluded, as they presented more severe polyneuropathies than diabetic patients taking oral antidiabetic drugs. Thirty-nine healthy volunteers without liver disease were also included. Exclusion criteria for all groups were acute or chronic pain and any signs of superficial inflammation or injury in the left foot or hand, to avoid interference with the sensitivity results. All participants were included after signing a written informed consent. Study protocols were in accordance with the ethical guidelines of the Declaration of Helsinki and were approved by the Research Ethics Committees of both hospitals (2018/210).

After performing psychometric tests, patients were classified as with or without MHE (see below) and were referred to the neurophysiology unit to undergo electrophysiological and quantitative sensory testing. These tests were performed within the following week after the PHES was performed, in order to minimize possible cognitive fluctuations. The composition and characteristics of the groups are given in Table 1.

### 2.2. Neuropsychological Assessment

MHE was diagnosed by the Psychometric Hepatic Encephalopathy Score (PHES) [13]. The global PHES scores were calculated with Spanish normality tables (http://www.redeh.org/TEST_phes.htm), adjusting for age and education level. Patients were defined as having MHE with a score ≤ −4 points.

To evaluate other cognitive abilities, further psychometric and motor tests were performed: the Stroop test (congruent, neutral, and incongruent tasks) for selective attention and cognitive flexibility; the d2 test, which evaluates selective/sustained attention and mental concentration; bimanual and visual-motor coordination tests; the Symbol Digit Modalities Test (Oral SDMT), for mental processing speed; Digit Span, evaluating immediate and working memory; and Letter-Number Sequencing, which evaluates working memory in greater depth than Digit Span. These tests were performed as in Giménez-Garzó et al. [14] on the same day as the PHES.

### 2.3. Neurophysiological Studies of Large Caliber Fibers: Nerve Conduction Study

The electrophysiological study of sensory and motor nerve conduction was undertaken with Synergy, version 22.0.0.144 and components: UltraPro S100 version 1 and UltraProS100 DSP version 591. Nerve conduction studies allowed us to determine the conduction of motor and sensitive fibers, including large and myelinated fibers, which have the greatest conduction velocity. This study allowed the detection and classification of the type of progressive polyneuropathy and to further identify the origin of sensory deficits.

Each laboratory should elaborate their own independent protocol of neurophysiological evaluation due to the differences that exist between testing equipment, techniques, and individual characteristics of the study population. The conduction study protocol used for the diagnosis of polyneuropathy was based on those described by Falck and Stålberg [28] and Preston and Shapiro [29].

The parameters measured were amplitude, latency, and conduction velocity. The latency measures nerve conduction time, in milliseconds (ms), from which the stimuli begins, to the initial moment of the evoked response; amplitude is the median value, in millivolts (mV), of the negative peak and positive peak of the evoked response, it assessed the number of stimulated axons; and conduction velocity, expressed in m/s, was calculated by measuring two stimulated points of the same nerve and dividing it by the difference between proximal latency and distal latency.

The sensory nerves explored were the unilateral ulnar, unilateral superficial radial, bilateral sural, and bilateral superficial peroneal nerves. The motor nerves examined were the unilateral ulnar, bilateral peroneal nerves, and bilateral posterior tibial nerves. Data obtained from nerve conduction measures were used to detect large nerve fiber damage, i.e., polyneuropathy. The diagnosis of polyneuropathy was based on the alteration of 3 or more nerves in 2 different extremities. See Supplementary Information for a more detailed explanation about the method used for nerve conduction studies.

### 2.4. Neurophysiological Studies of Small Caliber Fibers: Autonomous Nervous System and Quantitative Sensory Testing (QST)

To evaluate whether small caliber fibers are affected in MHE, we performed neurophysiological tests including study of the autonomous nervous system (sympathetic skin response, RR interval) and Quantitative Sensory Testing.

#### 2.4.1. Autonomous Nervous System

Sympathetic skin response (SSR) can be used to explore sudomotor function, which is part of the thermoregulation system [30]. SSR was analyzed using the Synergy UltraPro S100. Registering electrodes were placed on the right palm with a reference electrode on the back of the hand. The electrodes used were common to the EMG technique, with a low frequency filter of 0.5 Hz. The stimulus used was small electric stimulation, and the amplitude (in mV) and latency (in ms) of response were registered. The amplitude of SSR was considered abnormal when it was lower than 1.1 mV. This threshold was calculated from the mean of amplitude of sympathetic skin response of controls minus 2 standard deviations.

We also studied heart rate variability as a measure of the cardiovascular autonomic nervous system. We determined resting heart rate via the vagal tone, and heart rate variability, which consisted of variation of the RR interval of the electrocardiogram, by evaluating fluctuations in heart rate. Duration of RR intervals recorded over time reflects the influence of both sympathetic and parasympathetic nervous systems in heart rate modulation. Among the different activation maneuvers, we performed the Valsalva maneuver, hyperventilation, and orthostatic tests (from lying to sitting position) [31]. Registering electrodes were placed on both wrists above the radial artery.

#### 2.4.2. Quantitative Sensory Testing (QST)

QST was performed using CASE IV System WR Testworks (Supplementary Figure S1). Case IV was an automated diagnostic device, which detects and characterizes disease-altered sensory thresholds of sensory receptors, nerve fibers, central nervous system tracts, and/or cerebral association areas [32,33,34,35]. In this study, we assessed two somatosensory pathways: the leminiscal pathway, tested by way of vibration, and the ventrolateral pathway, evaluated through heat-pain and cooling perception. These thermal and nociceptive sensations were transmitted through small sensory nerve fibers [36], which can be classified by diameter and myelination. Vibration travels through large diameter sensory myelinated fibers (A alpha), while cooling perception mainly is relayed by small diameter myelinated fibers (A delta), and heat-pain is transmitted by unmyelinated (C fibers) [23]. The parameters registered were: vibration detection threshold (VDT), mediated by large diameter sensory myelinated fibers, A alpha; cooling detection threshold (CDT) measuring mainly small diameter myelinated fibers, A delta; and heat pain detection threshold (HPDT), mediated by small diameter unmyelinated C fibers. Supplementary Figure S2 shows the algorithm used, which determines how the stimuli are presented. The calculation of sensory thresholds is detailed in Supplementary Materials.

A total of six tests were performed: three modalities (VDT, CDT, and HPDT) on two test sites, hand and foot, in that order. Results were represented by the sensory thresholds of each individual test in just-noticeable difference (JND), corresponding to the mean stimuli level just detectable by the subject. A normal range was calculated from data collected from the control group, and tests outside the normal range were summed up for each individual patient. The total time to complete each test was measured in seconds.

### 2.5. Statistical Analysis

Values are given as mean ± standard error of mean (SEM), unless otherwise specified. D’Agostino and Pearson omnibus normality test was used to test variable normality. Between-group differences were analyzed using one-way ANOVA followed by post-hoc Tukey’s multiple comparisons test. For non-parametric variables the Kruskal-Wallis test was performed, followed by Dunn’s multiple comparisons test. Results were analyzed by GraphPad PRISM Version 7. The probability level accepted for significance was *p* < 0.05. Bimanual and visual-motor coordination tests were analyzed using univariate analysis of covariance (ANCOVA) with age included as covariate, followed by post-hoc Bonferroni. Analyses of contingency tables were performed by Fisher’s exact test. We evaluated the predictive capacity of QST parameters for MHE using ROC (receiver operating characteristic) curves. Pearson correlation analysis and ROC analyses were performed using the SPSS software, Version 20 (SPSS Inc, Chicago, IL, USA) and two-sided *p*-values < 0.05 were considered significant.

## 3. Results

### 3.1. Neuropsychological Assessment

After PHES, cirrhotic patients were stratified into 38 patients without MHE (NMHE) and 20 with MHE (Table 1).

Selective and sustained attention (measured by Stroop and d2 tests) and mental processing speed (by oral SDMT) were altered in both groups of patients compared to controls, but patients with MHE performed worse in these tasks than NMHE patients (Table 2).

Patients with MHE also showed impairment in working memory tasks such as Digit Span and Letter-Number Sequencing tests, performing worse than NMHE patients in the latter test (Table 2). Finally, bimanual, and visual-motor coordination were poorer in MHE patients than in NMHE patients (*p* < 0.001).

### 3.2. Nerve Conduction Study

**Figure 1:**
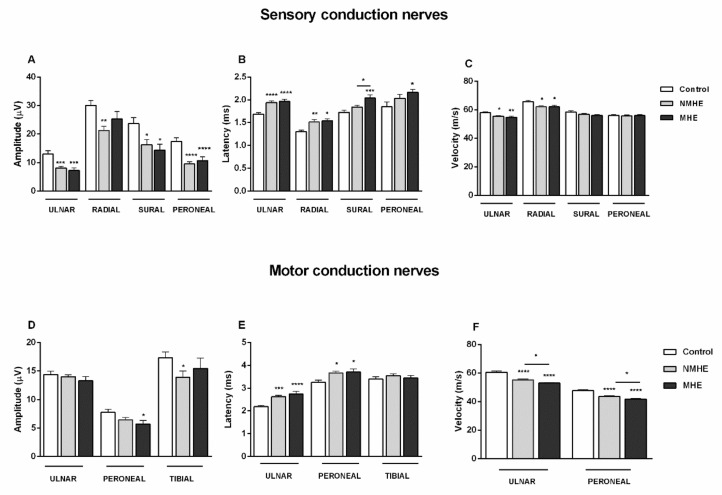
Cirrhotic patients with and without MHE show alterations in sensory and motor nerve conduction. (**A**–**C**): Sensory-nerve conduction. (**A**) Amplitude; (**B**) Latency; (**C**) Nerve conduction velocity. Data are for these sensory nerves: ulnar, radial superficial, sural, and superficial peroneal nerves. (**D**–**F**): Motor-nerve conduction. (**D**) Amplitude; (**E**) Latency; (**F**) Nerve conduction velocity. Data for ulnar, peroneal, and tibial motor nerves are shown. MHE, NMHE: patients with and without minimal hepatic encephalopathy, respectively. Results are mean ± SEM. * *p* < 0.05; ** *p* < 0.01; *** *p* < 0.001; **** *p* < 0.0001.

Based on nerve conduction studies, polyneuropathy was detected in 22 cirrhotic patients (38%): 14 without MHE (37% of NMHE patients) and eight with MHE (40% of MHE patients) (Table 1). Proportions of polyneuropathy between groups were not statistically different (Fisher exact test *p* > 0.99). Ten NMHE patients and four patients with MHE had mild to moderate sensory axonal polyneuropathy. Four patients from each group showed sensory-motor axonal neuropathy. There were two controls who presented mild large fiber neuropathy. In order to avoid possible bias in the results when comparing to controls, we included these controls in the analysis in Figure 1, as well in Table 3.

Figure 1 shows the results of the nerve conduction study. Nerve sensory amplitudes were reduced in cirrhotic patients in all nerves studied, and latencies in ulnar and radial nerves were increased compared to controls (Figure 1A,B). Sural nerve latency was increased in MHE compared to NMHE patients. Nerve conduction velocity was reduced in ulnar and radial sensory nerves in both patient groups (Figure 1C). Nerve motor amplitude was reduced in the peroneal nerve in MHE patients (Figure 1D), and latencies were increased in ulnar and peroneal nerves in cirrhotic patients (Figure 1E). Conduction velocity in these motor nerves was reduced in both patient groups compared to controls, being significantly lower in MHE than NMHE patients (Figure 1F).

### 3.3. Quantitative Sensory Testing (QST)

QST total results for each group are displayed in Table 3. We observed higher thresholds and longer detection times in NMHE patients compared to controls in all modalities, except for heat pain 5.0 thresholds in the foot, and detection times for vibration and cooling in the hand, and heat pain in the foot (Table 3). Patients with MHE showed higher thresholds than NMHE patients in cooling detection (*p* = 0.04), heat pain 0.5 (*p* = 0.01) and heat pain 5.0 (*p* = 0.04) when the foot was the test site (Table 3). The proportion of patients in which over half the tests were outside the normal range was higher (*p* = 0.007) in MHE (67%) than in NMHE patients (26%).

Control subjects finished each test in around 120 s, the NMHE group taking slightly longer (125–180 s) and the MHE group longer still (130–220 s). Significant differences between patients were found in vibration detection time (*p* = 0.04), cooling detection time (*p* = 0.01) and heat pain time (*p* = 0.01) when the foot was the test site (Table 3).

Regarding autonomic function, there was a general decrease in all parameters of heart rate variability (R-R interval), being significantly reduced in NMHE patients compared to controls in the hyperventilation test (*p* < 0.05). The amplitude of SSR was significantly reduced in patients with MHE compared to controls (*p* < 0.01) and NMHE patients (*p* < 0.05) (Table 3).

To test the influence of sex on QST parameters, we performed an analysis of QST parameters comparing results from by sex in the control group. Although there was a higher proportion of women in the control group than in the patient groups (Table 1), we did not find any significant differences between males and females in the variables analyzed (Supplementary Table S1).

### 3.4. Comparisons between Patients with and without MHE with Normal Sural Nerve Amplitude

We selected sural nerve amplitude as a representative value of the nerve conduction study, as it is the most distal nerve studied in lower limbs, and one of the first nerves to be affected in large fiber polyneuropathy. Sural nerve amplitude values were considered normal or pathologic according to reference values from our laboratory (cut-off: 15 µV). Sural nerve amplitude was affected in 42% of NMHE patients and 40% of patients with MHE, indicating an impairment of the large sensory fiber. Supplementary Table S2 shows the results of nerve conduction studies in controls and patients with normal sural nerve. Patients with MHE presented longer latencies than controls in ulnar and radial sensory nerves. Ulnar motor nerve latency was increased in both NMHE and MHE patients compared to controls, whereas the conduction velocities of ulnar and peroneal motor nerves were decreased in both groups of patients compared to controls, being significantly lower in MHE than in NMHE patients in ulnar motor nerve.

**Figure 2:**
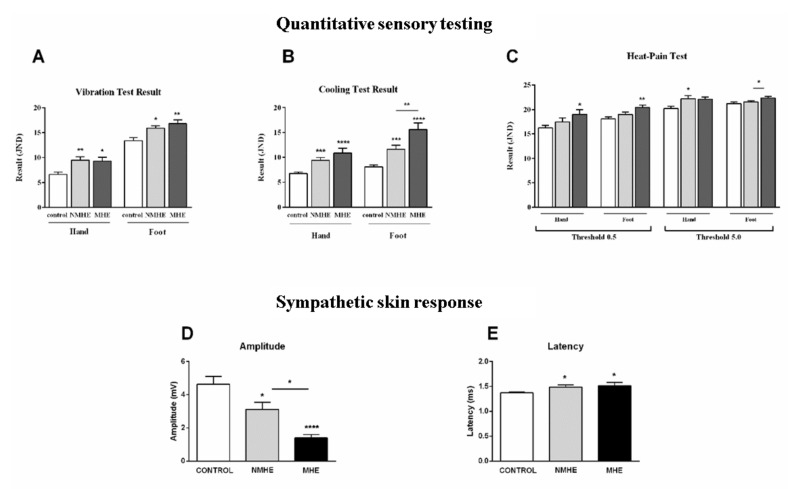
MHE patients with normal sural nerve amplitude show alterations in thermal sensitivity and sympathetic skin response compared to NMHE patients and controls. (**A**–**C**) Quantitative sensory test (QST) parameters: (**A**) Vibration detection threshold, (**B**) Cooling detection threshold, and (**C**) Heat Pain detection threshold, at 0.5 and 5.0 thresholds. (**D**–**E**) Sympathetic skin response: (**D**) Amplitude, (**E**) Latency. JND: just noticeable differences; MHE, NMHE: patients with and without minimal hepatic encephalopathy, respectively. Results are the mean ± SEM. * *p* < 0.05; ** *p* < 0.01; *** *p* < 0.001; **** *p* < 0.0001.

Looking for early markers of MHE based on neurophysiological parameters, we checked for signs of small caliber fiber alterations in patients without affected sural nerve amplitude, by assessing sympathetic skin response, RR interval variation, and QST (Table 4, Figure 2).

A greater affectation was found in patients with MHE compared to NMHE in the modality of conduction heat pain detection in the foot, with a trend towards significance (*p* = 0.06) in the sensitive sensory threshold value (0.5 JND) and a significant increase in the maximum value reached during the heat pain test (5.0 JND) (*p* = 0.04). Patients with MHE had higher thresholds in thermal detection in cooling when the foot was the test site (*p* = 0.007), and also took a longer time to detect temperature changes-foot cooling detection time-(*p* = 0.007) compared with NMHE patients (Table 4; Figure 2). Moreover, 54% of MHE patients had abnormal scores in more than half of the QST tests performed, while for NMHE patients, the percentage was 28%.

There were no statistically significant differences between NMHE and MHE patients in vibration detection, but both cirrhotic patient groups had higher thresholds than controls in the two test sites (foot and hand). The same result was found for cooling detection when the hand was the test site (Table 4; Figure 2).

When analyzing autonomous functions among patients with normal sural nerve amplitude (without large fiber impairment), we found greater alterations in MHE than in NMHE patients, reflected in a decrease in heart rate variability (RR interval) (small fiber involvement) both in the baseline study at rest (*p* = 0.007) and in the orthostatic test or passive tilt test (*p* = 0.04) (Table 4).

Sympathetic skin response amplitude was abnormal in 36% of MHE patients and 11% of NMHE patients. Patients with MHE showed lower response potential in amplitude than NMHE patients (*p* = 0.03) (Table 4, Figure 2). Both NMHE and MHE patients showed lower amplitudes (*p* < 0.05, and *p* < 0.0001, respectively) and longer latencies (*p* < 0.05) than controls (Table 4; Figure 2).

We also tested the possible contribution of alcoholic etiology to the effects observed. We grouped the patients with alcoholic etiology together with those with mixed etiologies such as NASH + alcohol and virus + alcohol, to determine if there were differences between past alcohol abuse and other etiologies. There were no differences between patients with alcoholic-induced cirrhosis and those of other etiologies in the parameters analyzed in quantitative sensory system and autonomic studies (Supplementary Table S3).

Regarding the relationship between neuropathy and liver disease severity, no patients in this study were in Child Pugh C grade; indeed, 74% were in Child-Pugh A grade and 26% in B. We found no significant differences in QST parameters and autonomic testing when grouping according to liver disease severity (Supplementary Table S4).

Given that diabetes was another possible confounding factor, we grouped patients with normal sural nerve amplitude by presence or absence of this condition. Analysis of QST and autonomic variables showed no significant differences between patients with and without diabetes (Supplementary Table S5).

### 3.5. Correlations between QST Parameters, Autonomic System, and Psychometric Tests

We analyzed whether changes in QST parameters correlated with performance in psychometric tests by assessing different cognitive abilities (Table 5), finding vibration detection time in foot and hand, cold detection time in hand, and heat pain detection 0.5 in foot to be correlated with the PHES score.

Neutral and incongruent tasks from the Stroop test assessing cognitive flexibility and selective attention showed good correlations with QST parameters, especially with cooling detection threshold (hand: *r* = −0.473, *p* < 0.001; foot: *r* = −0.370, *p* = 0.002, vs. incongruent task).

Performance in d2 test, assessing selective and sustained attention and concentration was associated with time duration in vibration (hand), cooling (foot), and heat pain (foot) tests. Significant correlations were also found between performance in d2 test and thresholds in vibration (foot) and cooling in both hand and foot (Table 5).

Mental processing speed, measured by Oral SDMT, correlated with time duration in all QST tests, except for vibration detection time in hand and heat pain detection time in foot. This test also correlated with cooling detection threshold, in both hand and foot (Table 5).

Working memory, measured by Digit Span and Letter-Number Sequencing tests, was associated with cold detection time in foot and heat pain detection time in hand. Vibration and cooling thresholds in both hand and foot, and heat pain detection 5.0 in hand also correlated with working memory tests.

Motor coordination tests showed weak correlations with QST parameters.

There were significant correlations between sympathetic skin response amplitude and PHES score (*r* = 0.43; *p* = 0.02), and Stroop congruent and neutral tasks (*r* = 0.441; *p* = 0.027, and *r* = 0.51; *p* = 0.01, respectively).

Sympathetic skin response amplitude correlated with vibration threshold in foot (*r* = −0.361; *p* = 0.018) and cooling threshold in hand (*r* = −0.380; *p* = 0.013), and also with heat pain detection 0.5 in foot (*r* = −0.317; *p* = 0.049).

### 3.6. Predictive Capacity of QST Parameters for MHE

We performed a ROC analysis, and found that cooling detection in the foot had a significant predictive capacity for detecting MHE, with the following area under the ROC curve (AUC): Cooling detection in foot (JND): AUC: 0.739; *p* = 0.017; 95% CI (0.543–0.936) and for Cooling detection time in foot (s): AUC: 0.820; *p* < 0.001; 95% CI (0.543–0.936).

## 4. Discussion

The main findings of this study are: (a) Hyposensitivity is present in cirrhotic patients but is higher in patients with MHE; (b) MHE patients have impaired thermal sensitivity in the foot, both in cooling and heat pain detection; (c) This impairment correlates with worse performance in attention, mental processing speed, and working memory tests; (d) Thermal sensitivity and autonomic function, involving small caliber nerve fibers, are early alterations associated with MHE, which appear before sural nerve amplitude (large nerve fiber) is altered.

To our knowledge, the present results provide first time evidence of impaired detection of cold and temperature changes in MHE patients compared to those without MHE. Although lower sensitivity to vibration and thermal stimuli was observed in the two patient groups compared to the control group in both hand and in foot, MHE patients had greater hyposensitivity when the foot was the test site.

The different results found in lower limbs and upper limbs is explained because toxic-metabolic polyneuropathies such as those derived from liver diseases, are characterized by length-dependent involvement, therefore the lower distal limbs are the first to be affected. Alterations reported in hepatic encephalopathy are included within the chronic axonal polyneuropathies of toxic-metabolic origin, characterized by length-dependent dying-back degeneration of axons, initially affecting the sensory fibers and with a pattern of distal predominance. In nerve conduction studies, the main finding is a decrease of the SNAP (sensory nerve action potential) and CMAP (compound muscle motor action potential) amplitudes, affecting the lower extremities more, with normal conduction velocity and distal latency, although conduction velocity can be reduced to 70–80% of the reference values if there is a decrease in CMAP amplitude greater than 50%, associated to a selective loss of fast-conducting fibers [37].

Our results are in contrast with a previous study from Brenner et al. [24] where the hand was used as the test site, the result of which found impaired thermal perception in grade 2 HE patients, but no differences in MHE patients. They concluded that these alterations emerge mainly in advanced stages of the disease. Like Brenner et al., we also found no differences between the two patient groups when the test was performed on the hands. However, by testing the foot we found greater hyposensitivity in MHE patients, suggesting that in these patients the distal sensory fibers become impaired earlier than the proximal ones found in the hands, which don’t deteriorate until more advanced grades of HE [24].

The differences found between hand and foot test sites indicate that the foot is more sensitive in distinguishing between cirrhotic patients with or without MHE, while the hand seems to have more sensitivity to detect cirrhotic patients who have not yet developed MHE.

Peripheral neuropathy is a frequent complication of liver cirrhosis [19,21,22], potentially affecting more than 70% of cirrhotic patients; in our study, however, only 38% of cirrhotic patients presented peripheral neuropathy. This lower incidence could be due to differing cirrhotic patient profiles, given that previous studies found this high incidence in patients with end-stage liver disease awaiting liver transplantation [19], whereas patients in the present study all had compensated cirrhosis. Moreover, neuropathy grade has been linked to liver disease severity, with a higher neuropathy score in Child-Pugh class C patients than those in A and B [19]; in this study, nonetheless, no patients were in Child Pugh C grade, in fact most were in Child-Pugh A (74%), so we can reasonably rule out liver disease severity as underpinning the effects observed in this study.

Patients with liver disease are at high risk of developing other metabolic syndromes such as diabetes, in which small fiber neuropathy is often concomitant. In this study we show that most alterations observed in small fibers were not influenced by diabetes.

The studies of the autonomic nervous system (RR interval, sympathetic skin response) and of thermal sensitivity are neurophysiological studies useful for evaluating function of small fibers.

The term small fiber neuropathy refers to a group of neuropathies characterized by a selective or predominant disorder of the poorly myelinated peripheral A delta afferent fibers and the unmyelinated C fibers [38]. In the somatosensory nervous system, these fibers transmit information on temperature, pain, and itching, and in the neurovegetative nervous system they are involved in sudomotor, thermoregulatory, cardiovascular, gastrointestinal, urogenital, and other functions [39].

In this study we show that MHE is more associated with alterations in small fiber function (cooling and heat pain thresholds) rather than large fiber function (vibration threshold).

Patients with MHE with normal sural conduction do not present alterations in large nerve fibers; however, in our study, we found that they present alterations in thermal sensitivity and autonomic function, which imply alterations in small nerve fibers, which are involved in these functions. This would indicate that in MHE patients, alterations in thermal perception and autonomic nervous system precede alterations in large fibers, given the impairment in QST parameters and RR interval variation found in MHE patients compared to NMHE patients.

The autonomic or vegetative nervous system is part of the central and peripheral nervous system involved in regulating involuntary functions of the organism, maintaining internal homeostasis and adapting responses to variations in the external and internal environment. The sudomotor function is part of the thermoregulation system, a complex homeostatic system integrated in the hypothalamus. By studying this system we can assess possible abnormalities in autonomic nervous system function [30]. Within the patient group with normal sural nerve amplitude (no distal large fiber impairment) sympathetic skin response amplitude was altered in 36% of MHE patients compared to 11% in NMHE patients. MHE patients also presented more changes in heart rate variability (RR interval) (small fiber involvement) in both the baseline study at rest, and the orthostatic test or passive tilt test. These data indicate involvement of both the sympathetic and parasympathetic nervous systems, although with greater influence on the latter, in MHE.

Taken together, these results suggest that the small fibers of the autonomic nervous system (sympathetic skin response, RR interval, and thermal sensitivity) are altered in early stages of MHE while changes to the large sensory fibers (sural nerve) take place later on.

The correlations found indicate that deficits in attention, mental processing speed, and working memory are associated with an impaired QST response. Although the PHES was defined as the gold standard to detect MHE [12,13], it was later shown that it fails to detect certain mild cognitive alterations and classifies patients with these alterations as NMHE [14,15,16,17]. The fact that NMHE patients also presented significant impairments in QST parameters compared to controls could be associated with presence of early attention impairment undetected by PHES [14,15].

It is difficult to ascertain whether impairment is central or peripheral: both components are likely to be altered. The correlation with the Stroop test suggests that the attention needed to synchronize activity between high- and low-order areas of the parietal cortex to enhance relevant sensory signals may be deficient. This points to central impairment, in agreement with previous studies in which patients with overt HE showed alterations in thermal sensitivity strongly correlated with central impairment [25]. The significant correlations found between time detection in all QST modalities and attention tests performed could also be pointing not to a deficit in sensing (peripheral impairment), but to a deficit in reporting due to attention dysfunction (central impairment). Therefore, these results would indicate that the delayed response in thermal and vibration detection could be due to alterations in mental processing speed and attention.

The correlation of SSR amplitude with cooling and thermal thresholds might well reflect a problem with awareness level regulation due to presence of MHE.

MHE patients experience psychomotor slowing [17] and longer reaction times, which could contribute to the longer times needed by MHE than NMHE patients to perform the QST tests, mainly when the foot was the test site.

Cognitive impairment and autonomic dysfunction may share a common underlying pathologic mechanism. Impaired autonomic function is present in patients with mild cognitive impairment or several different types of dementia: Alzheimer’s disease, frontotemporal dementia, dementia with Lewy bodies, and Parkinson’s disease with dementia [40]. Mild cognitive impairment has been associated with orthostatic blood pressure dysregulation. Nicolini et al., [41] suggested that the underlying physiopathological mechanism could be the disruption of central autonomic control due to damage of the right insula or locus coeruleus damage. The insular cortex is involved in thermosensory function and pain [42,43]. In a previous study by magnetic resonance we found that MHE patients showed gray matter reduction in the right insula, which correlated with PHES score, attention tests, and inflammation [44]. This alteration could contribute to the thermosensory and autonomic impairments associated with MHE.

Quantitative sensory testing of cooling detection in the foot (both JND and detection time) has a significant predictive capacity for detecting MHE, as indicated by the obtained ROC curves. Although we cannot discern whether the results of cooling detection time were due to a peripheral deficit in “sensing” or to a central deficit in “reporting” due to the attention dysfunction, we consider this parameter to be of value in clinical practice and a useful element to consider in the diagnosis of MHE, because although it does not pin point the origin of the pathophysiology, it does seem to predict the presence of MHE. QST testing also gives us a more comprehensive way of evaluating MHE, allowing an easy, low cost, and non-invasive way to check on a patient’s evolution and incrementing opportunities to alter prognosis early on.

A limitation of this study was that there are no ‘pure’ cirrhotic patients, because they usually present other comorbidities, such as diabetes mellitus, arterial hypertension, kidney complications… MHE patients have central alterations, peripheral neuropathy (a frequent complication of liver cirrhosis) and a high probability of coinciding with other metabolic syndromes such as diabetes, which in turn, frequently present small fiber neuropathy. Consequently, it is difficult to differentiate whether the polyneuropathy present in cirrhosis is secondary to diabetes mellitus or cirrhosis. In this study, there were no significant differences between those with or without diabetes in early alterations of thermal sensitivity found in MHE patients, which could indicate a main role of MHE in these alterations.

Some medications, such as lactulose, could change the presence of MHE. As shown in Table 1, 3 NMHE patients (8% of total NMHE) and 4 MHE patients (20% of total MHE) were taking lactulose. Although it was reported in the literature that lactulose may improve MHE [45,46], this does not seem to be the case in our study, as four patients taking lactulose presented MHE.

Regarding the alterations in the autonomic function, it was also difficult to differentiate the contribution of liver cirrhosis from the use of medication for arterial hypertension, like beta-blockers. However, the differences in autonomic function observed between NMHE and MHE patients would not be due to beta-blockers, given that the proportion of MHE patients having this medication were lower (5/20; 25%) than NMHE patients (15/38; 39%)

## 5. Conclusions

In summary, patients with MHE experience a general decrease in cognitive and sensory abilities. The small fibers of the autonomic nervous system and cooling sensitivity are affected at early stages of MHE, predominantly distal and in the lower limbs, before the large sensory fibers become altered. We found early alteration in the sympathetic skin response in MHE patients, with lower prevalence in patients without MHE. This could be considered an early marker of pathophysiology, and could be useful for early detection of patients susceptible to developing MHE. Cirrhotic patients are much more likely to present a variety of comorbid syndromes such as diabetes and polyneuropathy, which worsen further prognosis. QST testing would help to detect these issues early on, as well as the presence of MHE, a point at which prognosis can still be altered. This early detection would allow patients to receive treatment and to improve their quality of life. Quantitative sensory tests could be a practical and functional clinical tool to use as a complementary test to detect MHE.

## Figures and Tables

**Table 1:** Composition of the different groups and etiology of liver disease.

	Control	NMHE Patients	MHE Patients
Number of subjects	39	38	20
Sex (male/female)	13/24	36/2	18/2
Age ^†^	64 ± 2	60 ± 1	64 ± 1
Etiology of cirrhosis			
Alcohol		18	10
HCV/HBV/HCV + alcohol		13/0/1	4/1/0
NASH/NASH + alcohol		1/3	4
other		2	1
Diabetes (without/DM NID)		31/7	12/8
Child Pugh score (A/B/C)		29/9/0	14/6/0
MELD score ^†^		9.4 ± 2.7	9.1 ± 2.3
Lactulose		3 (8%)	4 (20%)
Beta-blockers	2 (5%)	15 (39%)	5 (25%)
Polyneuropathy (no/yes) (%)	37/2 (95/5)	24/14 (63/37)	12/8 (60/40)

^†^ Values are expressed as mean ± SD. MHE, NMHE: patients with and without minimal hepatic encephalopathy, respectively; HBV: hepatitis B virus; HCV: hepatitis C virus; NASH: non-alcoholic steatohepatitis; DM NID: diabetes mellitus without insulin dependence; MELD: model end-stage liver disease.

**Table 2:** Performance in neuropsychological tests.

	Controls	NMHE Patients *p* vs. Control	MHE Patients *p* vs. Control	MHE Patients *p* vs. NMHE	Global ANOVA *p* Values
PHES Global score	0.0 ± 0.2	−0.8 ± 0.2	−7.5 ± 0.7 ***	<0.001	<0.001
Bimanual coordination (min)	2.2± 0.1	2.3 ± 0.1	3.4 ± 0.3 ***	<0.001	<0.001
Visual-motor coordination (min)	2.7 ± 0.1	2.9 ± 0.1	3.7 ± 0.2 ***	<0.001	<0.001
d2 Test					
TR Values	378 ± 19	322 ± 11 *	271 ± 14 ***	0.03	<0.001
TOT Values	366 ± 16	292 ± 12 **	245 ± 15 ***	0.05	<0.001
CON Values	146 ± 7	114 ± 6 *	89 ± 10 ***	0.04	<0.001
TA Values	142 ± 7	117 ± 6 *	98 ± 7 **	ns	0.001
Stroop test					
Congruent Task ^†^	103 ± 4	94 ± 3	81 ± 4 **	0.04	0.003
Neutral Task ^†^	78 ± 3	69 ± 2 *	57 ± 2 ***	0.001	<0.001
Incongruent Task ^†^	47 ± 2	37 ± 2 **	30 ± 2 ***	0.02	<0.001
Oral SDMT (correct pairings)	44 ± 2	38 ± 1 *	26 ± 2 ***	<0.001	<0.001
Digits Span-Total score	14 ± 0.7	12 ± 0.6 *	11 ± 0.7 **	ns	0.007
Letter-Number Sequencing test (right answers)	9 ± 0.6	7 ± 0.6	5 ± 0.7 **	0.02	0.001

Values are expressed as mean ± SEM. Differences between groups were analyzed using one-way ANOVA followed by post-hoc Tukey. For bimanual and visual-motor coordination tests, univariate analysis of covariance (ANCOVA) was performed, with age included as a covariate, followed by post-hoc Bonferroni. Significant differences compared to controls are indicated by asterisks: * *p* < 0.05; ** *p* < 0.01; *** *p* < 0.001. MHE and NMHE: patients with and without Minimal Hepatic Encephalopathy: respectively; PHES: Psychometric Hepatic Encephalopathy Score; d2 test: TR: Total number of characters processed; TOT: Total correctly processed; CON: Concentration performance; TA: Total right answers. † Stroop test: Congruent task: number of words read in 45 s; Neutral task: number of colors read in 45 s; Incongruent task: number of items completed in 45 s. Oral SDMT: Symbol digit modalities test (oral version).

**Table 3:** Comparison of QST parameters and autonomic testing in controls and cirrhotic patients.

QST Parameters		Controls	NMHE Patients *p* vs Control	MHE Patients *p* vs Control	MHE Patients *p* vs. NMHE	ANOVA Global *p* Values
Vibration detection (JND)	hand	7 ± 0.5	9 ± 0.5 **	10 ± 0.5 **	ns	<0.0001
	foot	13 ± 0.6	17 ± 0.4 ****	17 ± 0.6 ****	ns	<0.0001
Cooling detection (JND)	hand	7 ± 0.3	10 ± 0.5 ****	11 ± 0.8 ****	ns	<0.0001
	foot	8 ± 0.4	13 ± 0.7 ****	16 ± 1 ****	0.04	<0.0001
Heat pain 0.5 (JND)	hand	16 ± 0.5	18 ± 0.5 **	19 ± 0.7 **	ns	0.0006
	foot	18 ± 0.4	19 ± 0.3 **	21 ± 0.5 ****	0.01	<0.0001
Heat pain 5.0 (JND)	hand	20 ± 0.5	22 ± 0.4 **	22 ± 0.7 *	ns	0.004
	foot	21 ± 0.3	22 ± 0.2	23 ± 0.6 **	0.04	0.005
Vibration detection time (s)	hand	127 ± 1	132 ± 2 *	138 ± 3 **	ns	0.004
	foot ^†^	127 ± 2	132 ± 2	141 ± 4 ***	0.04	0.0007
Cooling detection time (s)	hand ^†^	141 ± 1	154 ± 5	155 ± 3 **	ns	0.004
	foot ^†^	144 ± 2	176 ± 8 **	213 ± 15 ****	0.01	<0.0001
Heat pain time (s)	hand	115 ± 8	182 ± 11 ***	189 ± 18 ***	ns	0.0001
	foot	125 ± 8	149 ± 9	187 ± 14 ***	0.01	0.0009
**Autonomic testing**						
R-R Interval variation (%)	Basal ^†^	3.2 ± 0.7	3.1 ± 0.5	2.0 ± 0.2	ns	0.264
	Hyperventilation	11.4 ± 2.0	5.9 ± 0.8 *	7.1 ± 1.8	ns	0.021
	Valsalva	15.6 ± 2.1	9.3 ± 1.3	9.9 ± 2.0	ns	0.389
	Orthostatic test	8.2 ± 2.5	5.5 ± 1.0	3.5 ± 0.7	ns	0.130
Sympathetic skin response	Amplitude	4.1 ± 0.6	2.6 ± 0.3	1.5 ± 0.3 **	0.047	<0.001
	Latency	1.40 ± 0.04	1.57 ± 0.04 **	1.59 ± 0.06 *	ns	0.015

Values are expressed as mean ± SEM. Between-group differences were analyzed using one-way ANOVA followed by post-hoc Tukey’s multiple comparisons test, with the exception of non-parametric variables, (^†^) in which Kruskal-Wallis test followed by Dunn’s multiple comparisons test was performed. Differences compared to control group are indicated by asterisks: * *p* < 0.05; ** *p* < 0.01; *** *p* < 0.001; **** *p* < 0.0001. QST: quantitative sensory test; MHE, NMHE: patients with and without minimal hepatic encephalopathy, respectively; s: seconds, JND: just noticeable differences.

**Table 4:** Comparison of QST parameters and autonomic testing in patients with normal sural nerve amplitude.

*QST* Parameters		Controls	NMHE Patients *p* vs. Control (*n* = 18)	MHE Patients *p* vs. Control (*n* = 11)	MHE Patients *p* vs. NMHE	ANOVA Global *p* Values
Vibration detection (JND)	hand	7 ± 0.5	9.5 ± 0.7 **	9.3 ± 0.7 *	ns	0.0008
	foot	13 ± 0.6	16 ± 0.5 *	17 ± 0.8 **	ns	0.002
Cooling detection (JND)	hand	7 ± 0.3	9.4 ± 0.6 ***	11 ± 1 ****	ns	<0.001
	foot	8 ± 0.4	11 ± 1 ***	15 ± 1.4 ****	0.004	<0.001
Heat pain 0.5 (JND)	hand	16 ± 0.5	17.5 ± 0.8	19 ± 1 *	ns	0.04
	foot	18 ± 0.4	19 ± 0.5	20 ± 0.5 **	0.06	0.01
Heat pain 5.0 (JND)	hand	20 ± 0.5	22 ± 0.6 *	22 ± 0.4	ns	0.013
	foot	21 ± 0.3	21 ± 0.2	22 ± 0.3	0.04	0.04
Vibration detection time (s)	hand	127 ± 1	132 ± 3	135 ± 3 *	ns	0.04
	foot ^†^	127 ± 2	134 ± 3 *	137 ± 2 **	ns	0.001
Cooling detection time (s)	hand ^†^	141 ± 1	149 ± 7	156 ± 5 *	ns	0.014
	foot ^†^	144 ± 2	156 ± 6	205 ± 22 ****	0.007	<0.001
Heat pain time (s)	hand	115 ± 8	166 ± 16 **	167 ± 20 *	ns	0.004
	foot	125 ± 8	153 ± 13	160 ± 16	ns	0.06
**Autonomic testing**						
RR Interval variation (%)	Basal ^†^	3.7 ± 0.7	4.3 ± 0.9	1.8 ± 0.1 *	0.007	0.008
	Hyperventilation	9.5 ± 1.4	6.7 ± 1.3	6.8 ± 2	ns	0.15
	Valsalva	12 ± 2	11 ± 2	10 ± 3	ns	0.95
	Orthostatic test	5 ± 1	6.9 ± 1.4	2.6 ± 0.4	0.04	0.05
Sympathetic skin response	Amplitude	4.6 ± 0.5	3.1 ± 0.4 *	1.4 ± 0.2 ****	0.03	0.0001
	Latency	1.4 ± 0.02	1.5 ± 0.03 *	1.5 ± 0.06 *	ns	0.04

Values are expressed as mean ± SEM. Between-group differences were analyzed using one-way ANOVA followed by post-hoc Tukey’s multiple comparisons test, with the exception of non-parametric variables, (^†^) in which Kruskal-Wallis test followed by Dunn’s multiple comparisons test was performed. Differences compared to control group are indicated by asterisks: * *p* < 0.05; ** *p* < 0.01; *** *p* < 0.001; **** *p* <0.0001. QST: quantitative sensory test; MHE, NMHE: patients without and with minimal hepatic encephalopathy, respectively; s: seconds, JND: just noticeable differences.

**Table 5:** Correlations of QST parameters with psychometric tests.

*QST* Parameters		PHES Score	Stroop Test	d2 Test		Oral SDMT	Digit Span	Letter-Number Sequencing	Coordination Tests
				d2-TOT	d2-CON				
Vibration detection (JND)	hand						−0.23 (0.06)	−0.40 (0.002)	
	foot		−0.31 (0.01)	−0.27 (0.03)	−0.26 (0.04)	−0.23 (0.06)	−0.39 (0.001)	−0.29 (0.02)	0.23 (0.07) ^§^
Cooling detection (JND)	hand	−0.33 (0.004)	−0.47 (<0.001)		−0.31 (0.01)	−0.36 (0.002)	−0.25 (0.04)	−0.38 (0.002)	0.23 (0.05)
	foot		−0.37 (0.002)	−0.25 (0.04)	−0.28 (0.02)	−0.36 (0.002)	−0.29 (0.02)	−0.30 (0.02)	
Heat pain 0.5 (JND)	hand		−0.21 (0.08)	−0.25 (0.04) ^‡^		−0.27 (0.03)			
	foot	−0.34 (0.005)	−0.22 (0.07)						0.29 (0.02) ^§^
Heat pain 5.0 (JND)	hand					−0.24 (0.06)	−0.32 (0.01)	−0.28 (0.03)	
	foot								
Vibration detection time (s)	hand	−0.32 (0.008)	−0.30 (0.02)	−0.34 (0.006)	−0.34 (0.009)			−0.25 (0.06)	0.24 (0.06) ^§^
	foot	−0.35 (0.003)	−0.24 (0.05)			−0.25 (0.06)			0.24 (0.06) ^§^
Cooling detection time (s)	hand	−0.22 (0.06)	−0.26 (0.03)	−0.26 (0.04) ^‡^		−0.38 (0.001)			0.25 (0.04)
	foot	−0.24 (0.04)	−0.29 (0.01)	−0.31 (0.01)	−0.32 (0.009)	−0.34 (0.004)	−0.41 (0.001)	−0.40 (0.001)	
Heat pain time (s)	hand		−0.25 (0.04) ^†^		−0.24 (0.05)	−0.33 (0.006)	−0.34 (0.006)	−0.30 (0.02)	
	foot			−0.29 (0.02)	−0.29 (0.02)				

The *r* values (*p*-values) of the Pearson correlation analysis are shown. Significant and near-significant correlations are shown. Values for Stroop test are for Incongruent task, except for (^†^), which are for Neutral task. ^‡^ Values for d2-TR, Total number of characters processed; ^§^ values for visual-motor coordination test; values for bimanual coordination test. QST: quantitative sensory test; JND: just noticeable differences; s: seconds; PHES: Psychometric Hepatic Encephalopathy Score; d2 test: TOT: Total correctly processed; CON: Concentration performance; Oral SDMT: Symbol digit modalities test (oral version).

## Data Availability

Data is contained within the article and supplementary material.

## Supplementary Materials

The following are available online at https://www.mdpi.com/2077-0383/10/2/239/s1, Supplementary methods: Quantitative Sensory Testing (QST); Neurophysiological studies of large fibers: nerve conduction study. Supplementary References. Figure S1. Quantitative Sensory Testing components; Figure S2. One—Time—Period 4, 2, 1 Stepping Algorithm; Table S1. QST and autonomic testing parameters comparing males and females in the control group. Table S2. Parameters of sensory and motor nerve conduction in controls and patients with normal sural nerve Table S3. Comparison of QST parameters and autonomic testing between patients with alcoholic etiology and with other etiologies, in the group of patients with normal sural nerve; Table S4. Contribution of liver disease severity to results observed in patients with normal sural nerve; Table S5. Comparison of QST parameters and autonomic testing in patients with normal sural nerve amplitude with and without diabetes.

## Abbreviations

HE	hepatic encephalopathy
MHE	minimal hepatic encephalopathy
PHES	psychometric hepatic encephalopathy score
SDMT	symbol digit modalities test
EMG	electromyography
QST	Quantitative Sensory Testing
SSR	sympathetic skin response
VDT	vibration detection test
CDT	cold detection test
HPDT	heat pain detection test
JND	just noticeable differences
NMHE	patients without MHE
