# Supplementary material for: Patients with Minimal Hepatic Encephalopathy Show Altered Thermal Sensitivity and Autonomic Function
Source: J Clin Med. 2021 Jan 11;10(2):239. doi: 10.3390/jcm10020239 (PMC7826803; doi:10.3390/jcm10020239)
Supplement: Supplementary file 1 [file jcm-10-00239-s001.pdf]

SUPPLEMENTARY MATERIAL

# Patients with Minimal Hepatic Encephalopathy Show Altered Thermal Sensitivity and Autonomic Function

**Dalia Rega [1,†], Mika Aiko [2,†], Nicolás Peñaranda [2,†], Amparo Urios [1,†], Juan-José Gallego [1], Carla Giménez-Garzó [3], Franc Casanova [1], Alessandra Fiorillo [1], Andrea Cabrera-Pastor [1], Teresa San-Miguel [4], Cristina Ipiens [2], Desamparados Escudero-García [5,6], Joan Tosca [5], Cristina Montón [5], María-Pilar Ballester [1,5], José Ballester [5], Luis Aparicio [7], María-Pilar Ríos [8], Lucía Durbán [8], Amparo Mir [6], Elena Kosenko [9], Paula Cases [2], Vicente Felipo [3] and Carmina Montoliu [1,4,*]**

**Contents:**

**Supplementary methods:**

- Quantitative Sensory Testing (QST)
- Neurophysiological studies of large fibers: nerve conduction study.

**Supplementary references.**

**Figure S1.** Quantitative Sensory Testing components

**Figure S2.** One – Time – Period 4, 2, 1 Stepping Algorithm

**Table S1**. QST and autonomic testing parameters comparing males and females in the control group.

**Table S2**. Parameters of sensory and motor nerve conduction in controls and patients with normal sural nerve.

**Table S3**. Comparison of QST parameters and autonomic testing between patients with alcoholic etiology and with other etiologies, in the group of patients with normal sural nerve.

**Table S4.** Contribution of liver disease severity to results observed in patients with normal sural nerve

**Table S5.** Comparison of QST parameters and autonomic testing in patients with normal sural nerve amplitude with and without diabetes

**Supplementary methods**

*Quantitative Sensory Testing (QST)*

The calculation of the sensory thresholds was done by administering a series of non-invasive vibratory or thermal stimuli, corresponding to a set of 25 standardized vibratory and thermal stimulation levels, according to a one-time-period 4, 2, 1 Stepping Algorithm (Fig. S2) ( *Dyck P, Zimmerman I, Gillen D, Johnson D, Karnes J, O'Brien P. Cool, warm, and heat-pain detection thresholds: Testing methods and inferences about anatomic distribution of receptors. Neurology. 1993; 43: 1500-1508*). This algorithm determines how the stimuli are presented. During a given test, subsequent stimuli may be dependent on a patient's response. These tests have a total of 20 stimulus trials; each trial corresponds to one-time period. During this time period, the stimulus may or may not be delivered (five periods of null stimuli are placed randomly to prevent false results). The green light will flash on the Patient Cue Device, signalling the beginning of a trial. Then, a "1" will be presented, signalling the time period. The subject must try to determine whether a stimulus (vibration or thermal) was delivered. The patient then answers by pressing "yes" or "no" on the Patient Response Device for cooling and vibration tests. For heat-pain testing the subject answers a number from 0 to 10, 0 being no pain and 10 being maximum pain possible.

The VDT stimuli are administered by the Vibration Stimulator (Fig. S1A) consisting of a galvanometer, set at 125 cycles per second, variable between 0 and 350 micrometres. The CDT and HPDT tests are done by the thermal Stimulator (Fig. S1B), a ceramic plate, held in place by a Velcro strap, which produces a specified temperature, which can be varied from 8.0 to 50.0 degrees C, with accuracy of 1.25 to 0.25 degrees C, on a 9.0-square-centimeter stimulating surface (traceable to National Institute of Standards and Technology, NIST, standards). Prior to testing, the Thermal Stimulator adjusts itself to match the patient's baseline skin temperature. For statistical normalization purposes, the baseline temperature was 30 ºC (for Cooling test) or 34 ºC (for Heat-Pain test). For high-magnitude thermal (warming) stimuli, a holding time is added to the waveform so that the absolute temperature is typically limited to 50ºC. The plateau lengthens the time that the stimulus is administered, providing more heat over time, ensuring the same physiologic sensation as a higher pyramidal-shaped waveform. For high-magnitude thermal (cooling) stimuli, the temperature is limited to 8ºC.

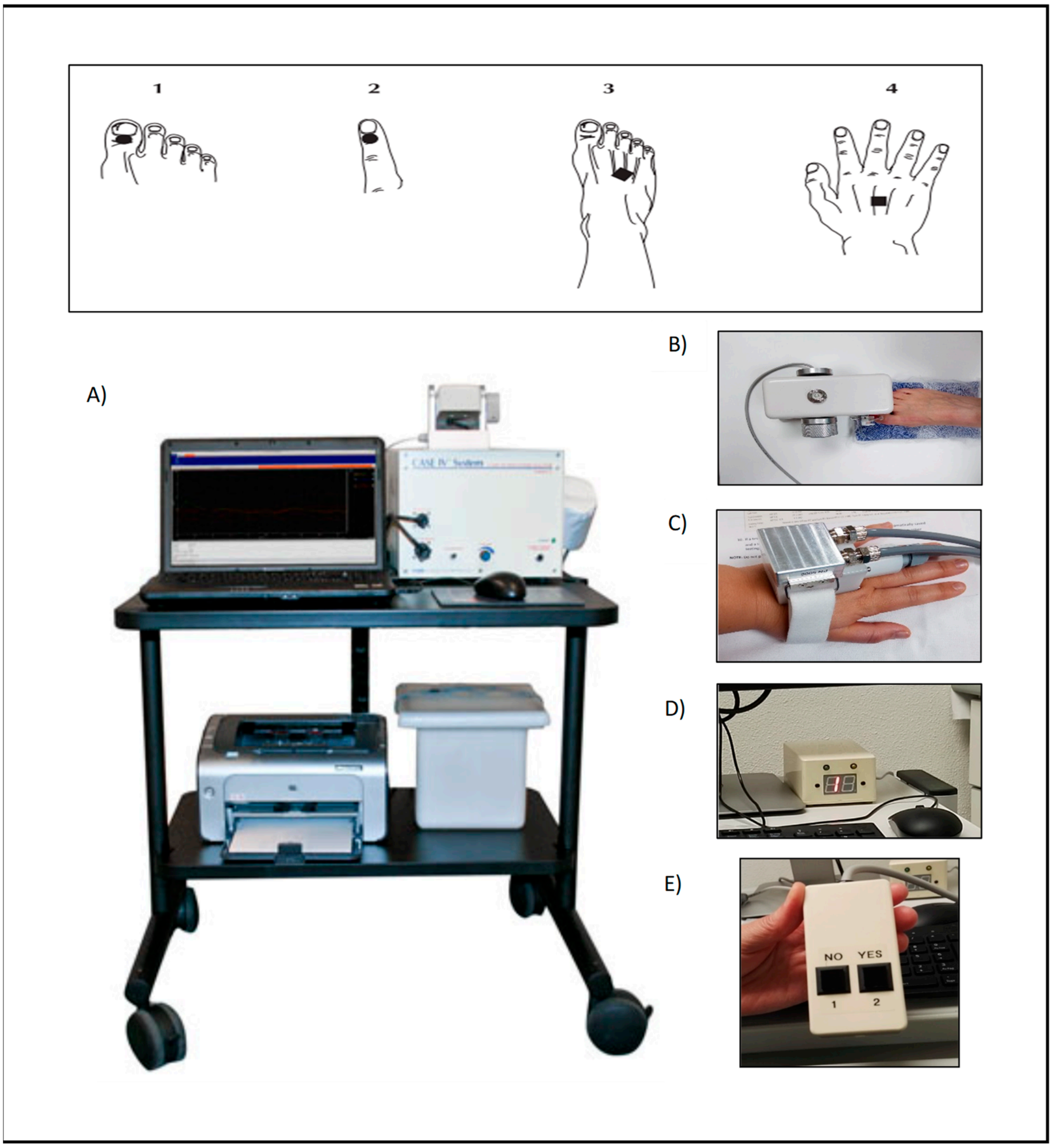


**Figure S1.** Quantitative Sensory Test components. The CASE IV system (**A**) consists of: The Vibration Stimulator (**B**), a galvanometer, set at 125 cycles per second, variable between 0 and 350 micrometres. The thermal Stimulator (**C**), a ceramic plate, held in place by a Velcro strap, which produces a specified temperature, which can be varied from 8.0 to 50.0 degrees C. These stimulus apparatuses are placed either on the hand or foot, 1 and 2 being for vibration detection tests and 3 and 4 being for thermal tests. The subject is then asked to pay attention to the box (**D**), when the light turns on they must be prepared to attend to the stimulus which is administered or not when the one appears. When the number turns off, the subject must respond if a stimulus was administered yes or no on the remote device (**E**).

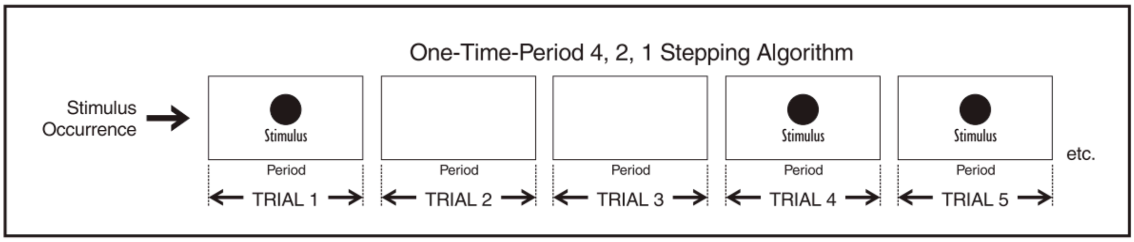


**Figure S2.** One – Time – Period 4, 2, 1 Stepping Algorithm. This algorithm determines how the stimuli are presented. During a given test, subsequent stimuli may be dependent on a patient's response. These tests have a total of 20 stimulus trials; each trial corresponds to one time period. During this time period, the stimulus may or may not be delivered (five periods of null stimuli are placed randomly to prevent false results) (modified form *Dyck P, et al. Cool, warm, and heat-pain detection thresholds: Testing methods and inferences about anatomic distribution of receptors. Neurology. 1993; 43:1500-1508*).

***Neurophysiological studies of large fibers: nerve conduction study***

Protocol: It is advisable that each laboratory should elaborate their own independent protocol of neurophysiological evaluation due to the differences which exist between testing machines, techniques, and individual characteristics of the study population.

The conduction study protocol used for the diagnosis of polyneuropathy, in this case, was based on those described by Stålberg [28] and Preston [29].

The studied sensory nerves were the:

- Unilateral ulnar.
- Unilateral superficial radial.
- Bilateral sural.
- Bilateral superficial peroneal nerves.

The motor nerves explored were the:

- Unilateral ulnar.
- Bilateral peroneal nerves.
- Bilateral posterior tibial nerves.

In addition, F waves of the unilateral cubital nerve and bilateral posterior tibial nerve were assessed. Also, autonomic testing was undertaken by assessing the R-R interval and cutaneous sympathetic response (CSR).

Reference values were also based on those described by Preston [29] and then adapted to our own normal values obtained from our laboratory and study population. These protocols [28] define that an axonal sensory polyneuropathy presents a series of characteristics: An amplitude reduction of sensory nerve conduction, with a greater affection of distal nerves in lower extremities (in more severe polyneuropathies motor nerve conduction amplitude may also be affected). Nerve conduction velocity is normal or can be slightly slower. F waves can have a delayed or diminished persistence. And autonomic tests are frequently altered.

Parameters: The parameters used to study evoked responses of nerve conduction were:

- Initial latency (measured nerve conduction time, in milliseconds, from which the stimuli begins, to the initial moment of the evoked response).
- Amplitude (median value, in millivolts, of the negative peak and positive peak of the evoked response, it informs us on the number of stimulated axons).
- Conduction velocity (expressed in m/s, calculated by measuring two stimulated points of the same nerve and dividing it by the difference between proximal latency and distal latency).

When studying nerve conduction parameters (sensory and motor) it was considered indicative of alteration when amplitude was diminished, conduction velocity was diminished, or latency was increased, according to established reference values *(Iriarte, F.; Artieda, J. Manual of Clinical Neurophysiology. Panamericana.* ***2012****, pp. 118-123).*

**Table S1.** QST and autonomic testing parameters comparing males and females in the control group

Values are expressed as mean ± SD. Differences between groups were analyzed by Student's T-test. Abbreviations: QST, quantitative sensory test; s, seconds, JND, Just noticeable differences.

| **QST parameters** | **Test site** | **Males** | **Females** | ***P*-values** |
|---|---|---|---|---|
| Vibration detection (JND) | hand | 6.0±0.9 | 7.0±0.5 | 0.332 |
| | foot | 13.0±1.0 | 13.2±0.9 | 0.887 |
| Cooling detection (JND) | hand | 6.7±0.5 | 6.8±0.4 | 0.864 |
| | foot | 7.9±0.4 | 8.3±0.6 | 0.660 |
| Heat pain 0.5 (JND) | hand | 15.5±1.0 | 16.6±0.8 | 0.377 |
| | foot | 17.2±0.8 | 18.2±0.5 | 0.252 |
| Heat pain 5.0 (JND) | hand | 20.2±0.9 | 19.9±0.7 | 0.792 |
| | foot | 21.5±0.6 | 21.2±0.5 | 0.748 |
| Vibration detection time (s) | hand | 128.56±2.9 | 127.16±1.5 | 0.637 |
| | foot | 126.5±2.6 | 128.0±2.6 | 0.712 |
| Cooling detection time (s) | hand | 143.9±2.0 | 138.8±1.6 | 0.060 |
| | foot | 144.0±3.1 | 143.6±3.3 | 0.942 |
| Heat pain time (s) | hand | 113.9±18.2 | 110.0±10.4 | 0.843 |
| | foot | 130.5±15.3 | 121.5±12.2 | 0.658 |
| **Autonomic testing** | | | | |
| R-R Interval variation (%) | Basal | 4.7±2.72 | 2.8±0.6 | 0.559 |
| | Hyperventilation | 18.2±4.9 | 9.3±1.7 | 0.163 |
| | Valsalva | 14.6±7.9 | 11.9±1.7 | 0.759 |
| | Orthostatic test | 14.5±8.1 | 6.2±2.21 | 0.176 |
| Cutaneous sympathetic response | Amplitude | 5.1±1.7 | 3.8±0.5 | 0.351 |
| | Latency | 1.33±0.02 | 1.42±0.05 | 0.362 |

**Table S2.** Parameters of sensory and motor nerve conduction in controls and patients with normal sural nerve.

Values are expressed as mean ± SEM. MHE, NMHE, patients with and without minimal hepatic encephalopathy,

| Parameters | Control | NMHE patients *P* vs. Control | MHE patients *P* vs. Control | MHE patients *P* vs. NMHE | ANOVA Global *P*-values |
|---|---|---|---|---|---|
| **Sensory nerve conduction** | | | | | |
| Ulnar sensory nerve amplitude (μV) | 13.1±1.4 | 9.8±0.6 | 10.0±1.0 | ns | 0.042 |
| Radial sensory nerve amplitude (μV) | 31.5±2.0 | 24.0±1.8* | 33.2±2.2 | 0.010 | 0.004 |
| Sural sensory nerve amplitude (μV) | 26.5±2.1 | 24.5±1.7 | 20.6±0.9* | ns | 0.059 |
| Superior peroneal amplitude (μV) | 18.2±1.3 | 13.3±1.3 | 15.5±0.7* | ns | 0.020 |
| Ulnar sensory nerve latency (ms) | 1.68±0.03 | 1.88±0.04** | 1.93±0.05** | ns | 0.001 |
| Radial sensory nerve latency (ms) | 1.28±0.03 | 1.43±0.05 | 1.50±0.05* | ns | 0.009 |
| Sural sensory nerve latency (ms) | 1.74±0.05 | 1.81±0.06 | 1.94±0.05 | ns | 0.092 |
| Superior peroneal latency (ms) | 1.92±0.06 | 2.05±0.08 | 2.14±0.08 | ns | 0.202 |
| Ulnar sensory nerve conduction velocity (m/s) | 58.1±0.8 | 56.3±0.8 | 54.6±0.7* | ns | 0.029 |
| Radial sensory nerve conduction velocity (m/s) | 66.0±1.1 | 62.4±1.0 | 61.9±1.3 | ns | 0.028 |
| Sural sensory nerve conduction velocity (m/s) | 58.9±1.1 | 58.2±0.9 | 56.5±1.1 | ns | 0.342 |
| Superior peroneal conduction velocity (m/s) | 56.5±0.8 | 56.4±0.9 | 55.9±1.0 | ns | 0.921 |
| **Motor nerve conduction** | | | | | |
| Ulnar motor nerve amplitude (μV) | 14.6±0.7 | 14.3±0.5 | 14.9±0.7 | ns | 0.799 |
| Peroneal motor nerve amplitude (μV) | 7.4±0.5 | 7.9±0.4 | 7.0±0.5 | ns | 0.475 |
| Tibial motor nerve amplitude (μV) | 17.4±1.1 | 15.6±1.0 | 18.8±1.9 | ns | 0.239 |
| Ulnar motor nerve latency (ms) | 2.18±0.06 | 2.52±0.07** | 2.69±0.17* | ns | 0.002 |
| Peroneal motor nerve latency (ms) | 3.29±0.11 | 3.56±0.09 | 3.52±0.16 | ns | 0.185 |
| Tibial motor nerve latency (ms) | 3.39±0.12 | 3.43±0.10 | 3.42±0.10 | ns | 0.953 |
| Ulnar motor nerve conduction velocity (m/s) | 61.3±1.0 | 56.2±1.0** | 53.1±0.4**** | 0.018 | <0.001 |
| Peroneal motor nerve conduction velocity (m/s) | 47.4±1.1 | 43.9±0.7* | 43.0±0.8** | ns | 0.004 |

respectively. Differences between groups were analyzed using one-way ANOVA followed by post-hoc Tukey's multiple comparisons test. Differences compared to control group are indicated by asterisks: * $p<0.05$; ** $p<0.01$; *** $p<0.001$; **** $p<0.0001$.

**Table S3**. Comparison of QST parameters and autonomic testing between patients with alcoholic etiology and with other etiologies, in the group of patients with normal sural nerve.

| | | Etiology | | |
|---|---|---|---|---|
| **QST parameters** | **Test site** | **alcohol** | **other** | ***P*-values** |
| Vibration detection (JND) | hand | 9 ± 4 | 9.7 ± 2 | 0.62 |
| | foot | 16.2 ± 2.3 | 16.5 ± 2.4 | 0.72 |
| Cooling detection (JND) | hand | 9.5 ± 3 | 10.5 ± 3 | 0.35 |
| | foot | 12.4 ± 5 | 13.2 ± 4 | 0.61 |
| Heat pain 0.5 (JND) | hand | 19.4 ± 3.5 | 17.4 ± 3 | 0.10 |
| | foot | 19.8 ± 1.5 | 19.7 ± 2 | 0.92 |
| Heat pain 5.0 (JND) | hand | 22 ± 2.4 | 22 ± 2.2 | 0.55 |
| | foot | 22 ± 1.2 | 22 ± 1 | 0.81 |
| Vibration detection time (s) | hand | 132 ± 10 | 134 ± 12 | 0.67 |
| | foot | 133 ± 7 | 136 ± 11 | 0.44 |
| Cooling detection time (s) | hand | 157 ± 34 | 148 ± 14 | 0.34 |
| | foot | 183 ± 80 | 170 ± 30 | 0.56 |
| Heat pain time (s) | hand | 163 ± 55 | 167 ± 77 | 0.87 |
| | foot | 165 ± 63 | 152 ± 46 | 0.55 |
| **Autonomic testing** | | | | |
| R-R Interval variation (%) | Basal | 3.6 ± 3.8 | 3.3 ± 2.7 | 0.80 |
| | Hyperventilation | 5.1 ± 3.7 | 8.2 ± 7 | 0.17 |
| | Valsalva | 11.4 ± 9.3 | 10.6 ± 7 | 0.79 |
| | Orthostatic test | 4.9 ± 5.2 | 5 ± 4.9 | 0.96 |
| Cutaneous sympathetic response | Amplitude | 1.9 ± 1.5 | 2.8 ± 1.6 | 0.13 |
| | Latency | 1.5 ± 0.2 | 1.5 ± 0.2 | 0.48 |

Values are expressed as mean ± SD. Differences between groups were analyzed by Student's T-test. QST, quantitative sensory test; s, seconds, JND, Just noticeable differences.

**Table S4**. Contribution of liver disease severity to results observed in patients with normal sural nerve

| | | Child-Pugh | | |
|---|---|---|---|---|
| **QST parameters** | **Test site** | **A** | **B** | ***P*-values** |
| Vibration detection (JND) | hand | 9.5 ± 2.8 | 8.9 ± 2.3 | 0.67 |
| | foot | 16.2 ± 2.3 | 17.1 ± 2.1 | 0.46 |
| Cooling detection (JND) | hand | 9.9 ± 3 | 10.5 ± 4 | 0.67 |
| | foot | 13.3 ± 4 | 10.8 ± 2 | 0.21 |
| Heat pain 0.5 (JND) | hand | 18 ± 3.5 | 19 ± 1.5 | 0.27 |
| | foot | 19.7 ± 2 | 19.8 ± 0.5 | 0.93 |
| Heat pain 5.0 (JND) | hand | 22.4 ± 2.4 | 21.6 ± 1 | 0.49 |
| | foot | 22 ± 1 | 22 ± 1.2 | 0.96 |
| Vibration detection time (s) | hand | 134 ± 11 | 130 ± 10 | 0.50 |
| | foot | 135 ± 10 | 133 ± 7 | 0.73 |
| Cooling detection time (s) | hand | 153 ± 26 | 151 ± 13 | 0.87 |
| | foot | 179 ± 61 | 157 ± 14 | 0.42 |
| Heat pain time (s) | hand | 168 ± 72 | 154 ± 45 | 0.69 |
| | foot | 154 ± 50 | 176 ± 77 | 0.46 |
| **Autonomic testing** | | | | |
| R-R Interval variation (%) | Basal | 3.2 ± 3 | 4.6 ± 5 | 0.47 |
| | Hyperventilation | 6 ± 4 | 11 ± 10 | 0.33 |
| | Valsalva | 11 ± 8 | 13 ± 9 | 0.56 |
| | Orthostatic test | 5 ± 5 | 4 ± 2 | 0.49 |
| Cutaneous sympathetic response | Amplitude | 2.6 ± 1.5 | 1.8 ± 2 | 0.36 |
| | Latency | 1.4 ± 0.2 | 1.7 ± 0.2 | 0.06 |

Values are expressed as mean ± SD. Differences between groups were analyzed by Student's T-test. Abbreviations: QST, quantitative sensory test; s, seconds, JND, Just noticeable differences.

**Table S5**. Comparison of QST parameters and autonomic testing in patients with normal sural nerve amplitude with and without diabetes.

| QST parameters | Test site | Without Diabetes | With Diabetes | *P*-values |
|---|---|---|---|---|
| Vibration detection (JND) | hand | 9.6 ± 3 | 9 ± 3 | 0.63 |
| | foot | 16 ± 2 | 15 ± 3 | 0.25 |
| Cooling detection (JND) | hand | 10 ± 3 | 10 ± 3 | 0.98 |
| | foot | 12.5 ± 4 | 13.7 ± 4 | 0.51 |
| Heat pain 0.5 (JND) | hand | 17.5 ± 3.4 | 20 ± 3 | 0.09 |
| | foot | 19.7 ± 2 | 19.2 ± 1 | 0.64 |
| Heat pain 5.0 (JND) | hand | 22 ± 2 | 23 ± 3 | 0.15 |
| | foot | 21.8 ± 1 | 22 ± 1 | 0.63 |
| Vibration detection time (s) | hand | 134 ± 11 | 131 ± 12 | 0.55 |
| | foot | 135 ± 10 | 133 ± 10 | 0.68 |
| Cooling detection time (s) | hand | 151 ± 26 | 153 ± 17 | 0.89 |
| | foot | 176 ± 59 | 171 ± 41 | 0.83 |
| Heat pain time (s) | hand | 162 ± 63 | 180 ± 78 | 0.55 |
| | foot | 163 ± 56 | 140 ± 38 | 0.33 |
| **Autonomic testing** | | | | |
| R-R Interval variation (%) | Basal | 3.3 ± 2.7 | 3.6 ± 4.5 | 0.85 |
| | Hyperventilation | 7 ± 6 | 5 ± 4 | 0.44 |
| | Valsalva | 10.6 ± 7 | 11 ± 10 | 0.90 |
| | Orthostatic test | 5.7 ± 5.3 | 2.4 ± 2 | 0.12 |
| Cutaneous sympathetic response | Amplitude | 2.9 ± 2 | 1.6 ± 1 | 0.10 |
| | Latency | 1.5 ± 0.2 | 1.5 ± 0.2 | 0.91 |

Values are expressed as mean ± SD. Differences between groups were analyzed by Student's T-test. Abbreviations: QST, quantitative sensory test; s, seconds, JND, Just noticeable differences.
